# Datasets of Bird Species Composition in a Land Reclamation Area of Lake Kahokugata, Central Japan, in Relation to Various Farmland Types

**DOI:** 10.1002/ece3.72039

**Published:** 2025-08-19

**Authors:** Masumi Hisano, Ken Motomura, Keinosuke Sannoh, Shota Deguchi

**Affiliations:** ^1^ Graduate School of Advanced Science and Engineering Hiroshima University Higashihiroshima Japan; ^2^ Graduate School of Informatics Kyoto University Kyoto Japan; ^3^ Graduate School of Agricultural and Life Sciences The University of Tokyo Tokyo Japan; ^4^ Board of Education, Nakano City Hall Nagano Japan; ^5^ Nihonkai Eco Engineering Technologies Toyama Japan; ^6^ Fukui City Museum of Natural History Fukui Japan

**Keywords:** artificial wetland, avian community, avifauna, bird species assemblage, passerine, shorebirds, waterfowl

## Abstract

Agricultural intensification and land reclamation have transformed natural wetlands into farmland across East Asia, which has been a threat to bird diversity, particularly wetland and grassland specialists. Despite extensive research in warm temperate and tropical rice‐growing regions, bird communities in snow‐rich agricultural wetland landscapes remain poorly studied. Here we present a dataset describing bird assemblages in a heterogeneous agricultural landscape surrounding Lake Kahokugata, located in a snow‐rich region on the Sea of Japan side of central Japan. The area represents a land reclamation zone shaped by decades of wetland conversion. We conducted point‐count surveys across 43 plots in winter and summer (2021 and 2023), yielding 129 replicated observations. The plots encompassed diverse cropland types, including rice paddies, lotus root fields, vegetable fields, pastures, and abandoned lands, representing a mosaic of wet and dry farmland. The dataset includes abundance records of 41 bird species, along with land‐use attributes within a 75 m radius and landscape data on open waterbodies within a 500 m radius. Our data is useful in providing insights into how landscape heterogeneity, cropland composition, and seasonal dynamics influence bird diversity in snowy agricultural wetlands.

## Graphical abstract

We present a dataset describing bird assemblages in a heterogeneous agricultural landscape surrounding Lake Kahokugata, located in a snow‐rich region on the Sea of Japan side of central Japan. Our data is useful in providing insights into how landscape heterogeneity, cropland composition, and seasonal dynamics influence bird diversity in snowy agricultural wetlands.

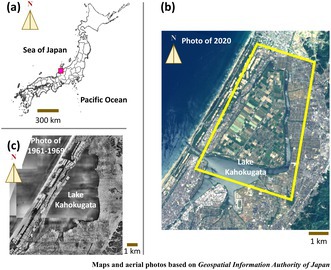

## 1 Introduction

Agricultural intensification has been the major threat to global bird diversity, driving the loss and degradation of their habitat (Rosenberg et al. 2019; Rigal et al. 2023). One of the most affected habitats is natural wetlands, which have been extensively converted into agricultural land through reclamation development over the past centuries (Wu et al. 2018; Fluet‐Chouinard et al. 2023; Kong et al. 2023). These transformations have led to global declines in grassland and wetland bird populations (Ma et al. 2019; Wang et al. 2020; Zhang et al. 2020), which rely on these habitats for nesting, foraging, and migration. Agricultural intensification has also led to reduced avian diversity (e.g., through taxonomic homogenization) and shifts in species, with wetland‐associated assemblages increasingly replaced by species typical of bare land or farmland (i.e., species turnover) (Sica et al. 2018; Kitazawa et al. 2022). In response to these threats, creating and managing artificial wetlands has been an expected strategy to restore wetland bird populations and diversity (Kačergytė et al. 2021; Cheng and Ma 2023). Nonetheless, agricultural landscapes must support not only biodiversity but also crop yields. In this context, there has been growing concern about how landscapes of agricultural land reclamation can be managed to conserve biodiversity (Yu et al. 2017, 2018) and ecosystem services (Zou et al. 2022) while maintaining crop productivity.

One of the landscape‐based approaches for biodiversity conservation in farmlands is the promotion of land use heterogeneity (Benton et al. 2003; Ricciardi et al. 2021). Heterogeneous landscapes with a mixture of crop types, non‐crop habitats, and semi‐natural vegetation are hypothesized to support greater avian diversity by providing a variety of resources and microhabitats (Benton et al. 2003; Hiron et al. 2015; Wilson et al. 2017). This concept has often been explored in studies from Western Europe (Hiron et al. 2015; Wuczyński 2016; Redlich et al. 2018) and North America (Rosin et al. 2016; Lee and Martin 2017; Wilson et al. 2017; Martin et al. 2020), where farmland heterogeneity and crop diversity have been shown to enhance bird diversity. Thus, by increasing habitat compositional diversity, niche availability, and retaining semi‐natural habitats, there is a growing insight that heterogeneous landscapes mitigate negative effects of agricultural intensification on bird diversity (Jeliazkov et al. 2016; Martin et al. 2020). However, our understanding of these dynamics remains limited in East Asia (Katayama et al. 2014; Liao et al. 2020; Lu et al. 2024), especially in the monsoon climate regions dominated by agricultural wetlands, including rice paddy systems (Amano et al. 2008; Katayama et al. 2021). This knowledge gap is critical given ongoing land reclamation and the intensification of agriculture at the expense of wetland ecosystems across East Asia (Hyun et al. 2013; Wu et al. 2018; Ma et al. 2019; Lee et al. 2020; Wang et al. 2020; Zhang et al. 2020).

In Japan, although many of the major rice‐producing areas in the country are located on the snow‐rich Sea of Japan side (MAFF 2024), most research on avifauna in rice paddies has been concentrated on the Pacific side with a snow‐poor climate (Kurechi 2007; Fujioka et al. 2010; Takeuchi 2019), especially in the inland areas of the Kanto region (Maeda 2001; Amano et al. 2008; Amano 2009; Katayama et al. 2015). In contrast, research on bird assemblages in land‐reclaimed rice paddies on the coastal Sea of Japan side, where heavy snowfall is common due to seasonal northwesterly winds, remains limited (Deguchi et al. 2020; Hisano et al. 2025). Globally, studies of bird use of rice paddies have primarily targeted non‐snowy temperate (Wood et al. 2010; Kim et al. 2013; Lee and Goodale 2018; Xie et al. 2019; Choi et al. 2022), subtropical (Chan et al. 2007; Lin et al. 2023), and tropical regions (Azman et al. 2011; Amira et al. 2018), where rice cultivation occurs under warm, moderate climates. In consequence, there is a critical lack of avifaunal data from snowy agricultural regions. This regional bias is further reflected in large‐scale citizen science programs in Japan [e.g., the Monitoring Sites 1000 project (Biodiversity Center of Japan 2024)], which tend to underrepresent areas along the Sea of Japan side. These disproportionate distributions of survey sites are likely due to higher population density and greater volunteer availability (Beck and Mitkiewicz 2025) on the Pacific side, which may introduce potential biases, such as confounding relationships or pseudo‐regression effects, resulting from the limited availability of detailed data in municipalities with smaller populations (De Coster et al. 2015). Therefore, accumulating bird data from the underrepresented is essential for detecting regional variation in avian responses to agricultural wetland habitats and for generalizing findings across Japan and East Asia. This is particularly important in snow‐rich regions, where patterns of snow cover can affect bird species distributions (Deguchi et al. 2022), as well as wintering and breeding timing and success (Liebezeit et al. 2014; Resano‐Mayor et al. 2019; Keyser et al. 2023). These snow‐related changes can also impact prey activity and availability, leading to phenological mismatches between birds and their food resources (Saalfeld et al. 2019; Wann et al. 2019). Moreover, ephemeral wetlands created by snowmelt in early spring can function as important stopover (Hisano et al. 2025) or breeding habitats (Li et al. 2020) for migratory birds.

**FIGURE 1:**
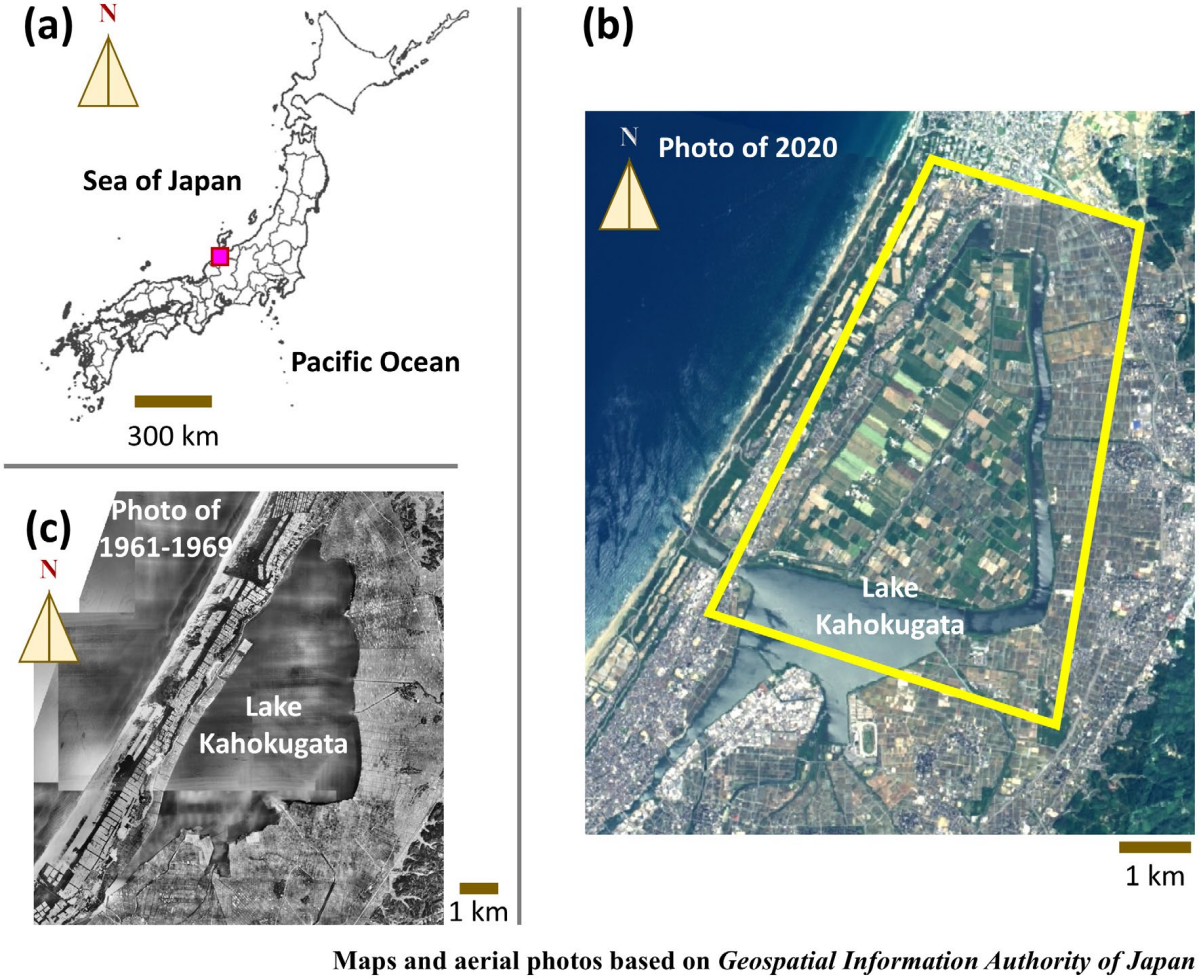
Maps showing the study area around Lake Kahokugata, central Japan. (a) Location of the study area (pink square) within the Japanese archipelago, located on the Sea of Japan side in Ishikawa Prefecture. (b) Aerial photograph from 2020 showing the current landscape of the study area (yellow rectangle), including the land reclamation area of Lake Kahokugata [reclamation completed in 1971 (Takahashi and Kawahara 2002)]. (c) Aerial photograph from 1961 to 1969 depicting the area before land reclamation. Maps and aerial images are based on materials provided by the Geospatial Information Authority of Japan (https://www.gsi.go.jp/).

Here we present data on bird species diversity and abundance in mosaic agricultural wetland landscapes surrounding Lake Kahokugata, central Japan, a snowy region on the Sea of Japan side (Figure 1a). The area exhibits a notable example of wetland transformation of agricultural land reclamation (Figure 1b,c). The reclaimed land was developed through the Kahokugata Reclamation Project (1963–1971), covering 1359 ha, of which 1079 ha are used for farming (Takahashi and Kawahara 2002). The reclaimed land also represents a cropland heterogeneity resulting from decades of cultivation land‐use change, including vegetable and grain fields, pastures, lotus root fields, rice paddies, fallow and abandoned fields, and non‐cropland areas (Takahashi and Kawahara 2002; Yamano 2012). This spatially and temporally dynamic fine‐scale cropland heterogeneity creates a mosaic of habitats that has the potential to support a wide variety of bird species. The dataset includes records of avian communities in and around the land reclamation area, along with qualitative and quantitative data on diverse agricultural land‐use patterns, reflecting landscape heterogeneity. This dataset has the potential to advance our understanding of how landscape heterogeneity and crop diversity influence avian diversity in understudied agricultural wetlands of snow‐rich regions in East Asia.

## 2 Data Description

### 2.1 Study Area

The study was conducted in the agricultural land reclamation area and its surrounding farmlands around Lake Kahokugata in Ishikawa Prefecture, central Japan (36°38′–36°42′N, 136°39′–136°42′ E). The mean annual temperature and mean annual precipitation of the area were 15.0°C and 2410 mm, respectively (average of 1991–2021); based on data from the Japan Meteorology Agency (2024).

### 2.2 Taxonomic Coverage

The dataset includes 41 bird species, which belong to 27 families and 11 orders (Table 1).

**TABLE 1:** List of bird species observed during point‐count surveys conducted in February**–**March 2021 and June 2023 in the area of Lake Kahokugata, central Japan.

Order	Family	Species	English common name	Migration type^a^
Accipitriformes	Accipitridae	*Buteo japonicus*	Eastern buzzard	Pv
		*Circus spilonotus*	Eastern marsh harrier	Rb
		*Milvus migrans*	Black kite	Rb
Anseriformes	Anatidae	*Anas platyrhynchos*	Mallard	Wv
		*Anas poecilorhyncha*	Spot‐billed duck	Rb
Charadriiformes	Charadriidae	*Charadrius dubius*	Little ringed plover	Mb
		*Vanellus cinereus*	Grey‐headed lapwing	Rb
		*Vanellus vanellus*	Northern lapwing	Wv
	Scolopacidae	*Gallinago gallinago*	Common snipe	Pv
Ciconiiformes	Ciconiidae	*Ciconia boyciana* ^b^	Oriental white stork^b^	Rb
Columbiformes	Columbidae	*Streptopelia orientalis*	Oriental turtle dove	Rb
		*Columba livia domestica* ^c^	Rock dove^c^	Rb
Cuculiformes	Cuculidae	*Cuculus canorus*	Common cuckoo	Mb
Galliformes	Phasianidae	*Phasianus colchicus*	Green pheasant	Rb
Gruiformes	Rallidae	*Gallinula chloropus*	Common moorhen	Rb
Passeriformes	Acrocephalidae	*Acrocephalus arundinaceus*	Great reed warbler	Mb
	Alaudidae	*Alauda arvensis*	Eurasian skylark	Rb
	Cettiidae	*Cettia diphone*	Japanese bush warbler	Rb
	Corvidae	*Corvus corone*	Carrion crow	Rb
		*Corvus macrorhynchos*	Large‐billed crow	Rb
		*Cyanopica cyanus*	Azure‐winged magpie	Rb
	Emberizidae	*Emberiza cioides*	Meadow bunting	Rb
		*Emberiza fucata*	Chestnut‐eared bunting	Mb
		*Emberiza rustica*	Rustic bunting	Wv
	Fringillidae	*Chloris sinica*	Oriental greenfinch	Rb
		*Carpodacus sibiricus*	Long‐tailed rosefinch	Wv
	Hirundinidae	*Hirundo rustica*	Barn swallow	Mb
	Laniidae	*Lanius bucephalus*	Bull‐headed shrike	Rb
	Motacillidae	*Motacilla alba*	White wagtail	Rb
		*Motacilla grandis*	Japanese wagtail	Rb
	Paridae	*Parus minor*	Japanese tit	Rb
	Passeridae	*Passer montanus*	Eurasian tree sparrow	Rb
	Pycnonotidae	*Hypsipetes amaurotis*	Brown‐eared bulbul	Rb
	Sturnidae	*Sturnus cineraceus*	White‐cheeked starling	Rb
	Sturnidae	*Sturnus vulgaris*	Common starling	Rb
	Turdidae	*Turdus naumanni*	Dusky thrush	Wv
	Zosteropidae	*Zosterops japonicus*	Japanese white‐eye	Rb
Pelecaniformes	Ardeidae	*Ardea alba*	Great egret	Rb
		*Ardea cinerea*	Grey heron	Rb
		*Egretta intermedia*	Intermediate egret	Mb
Piciformes	Picidae	*Dendrocopos kizuki*	Japanese pygmy woodpecker	Rb

Abbreviations: Pv, passage visitor; Rb, resident breeder; Wv, wintering visitor.

^a^ Based on the local report of the region of Lake Kahokugata by (Yamamoto et al. 2000) and the authors' prior knowledge (Hisano et al. 2025), supplemented by (Nakamura and Nakamura 1995; Higuchi et al. 1996, 1997).

^b^ Re‐introduced species.

^c^ Introduced species.

### 2.3 Methods

We conducted point‐count surveys from February 27th to March 1st, 2021 (the first survey); March 23rd to March 31st, 2021 [the second survey (Hisano and Deguchi 2021; Hisano et al. 2025)]; and June 12th to 15th, 2023 (the third survey). The first and second surveys covered the wintering season of farmland birds, while the third survey overlapped with their breeding season (Yamamoto et al. 2000). Given the lower abundance and detectability of wintering birds, which produce call notes but not songs, we conducted the winter surveys twice. A total of 43 plots were established on farm roads extending into the farmlands to collect the bird assemblage data. We employed point‐count methods by recording any bird individuals observed by sight and sound within a 75‐m radius circle for 10 min, including those flying over the plots. However, we carefully excluded individuals flying in from a direction already recorded a few minutes earlier (Deguchi et al. 2020; Hisano and Deguchi 2021; Hisano et al. 2025). To avoid cross‐site double counting, each site was spaced at least 300 m apart (Hiron et al. 2013).

The farmland types of the study sites were categorized as follows based on a cropland use map of 2003 and previous studies (Takahashi and Kawahara 2002; Yamano 2012), supplemented by the authors' direct visual observations in 2021 and 2023:

1. Vegetable and grain fields (e.g., soybeans, wheat, often bare land in winter);
2. Pastures (short height grasslands used for grazing cattle and producing fodder);
3. Lotus root fields (waterlogged fields throughout the year);
4. Rice paddies (temporally irrigated during summer, while drained in winter);
5. Fallow and abandoned fields (grasslands with various structures of vegetation or overgrown areas with reeds ( *Phragmites australis* ) and invasive tall weeds of Canadian goldenrods ( *Solidago canadensis* )); and
6. Non‐cropland areas (e.g., roads, buildings, shelterbelts, and open lands repurposed from agricultural use).

Since the plots were established along farm roads, the cropland types adjacent to each plot were identified for both sides of the road (either north/south or east/west), as the farm roads extend into the farmlands. Considering the two adjacent cropland types, the plots were further grouped into three farmland type combinations: “dry/dry” (a combination of dry croplands, including vegetable and grain fields, pasture, and/or fallow/abandoned fields), “wet/wet” (a combination of agricultural wetlands, including lotus root fields and/or rice paddies), and “dry/wet” (a combination of the above dry and wet cropland types; Table 2).

**TABLE 2:** Column names and definitions for the avifaunal datasets “all_data.csv” (including all recorded individuals, including flyovers) and “ground_data.csv” (excluding flyovers).

Column name	Definition	Unit or notes
id	Serial number (for ordering purposes)	
SITE	Study sites ID: B1‐B18 (sites with shelterbelts), O1‐O25 (open sites)	
Date	Year/Month/Date (yyyy/mm/dd)	
LAT	Decimal latitude	Degree
LONG	Decimal longitude	Degree
Greenbelt	Whether the site includes shelterbelts (“1”) or not (“0”) within a 75‐m radius from the centre of the site.	
Time	Time (hh:mm:ss) started the point‐count survey (for 10 min)	
SITE_MS	SITE and Date (yyyy_mm_dd)	
Session	Study session defined as “First” = Feb 27th‐Mar 1st, “Second” = Mar 23rd‐31st, “Third” = Jun 12th‐15th	
LandType	Land use type defined as “DD” = sites only include dry croplands (vegetable/grain fields, pastures, and/or fallow/abandoned fields), “WW” = sites only include agricultural wetlands (rice paddies and/or lotus fields), “DW” = sites including both dry croplands and agricultural wetlands, within a 75‐m radius	
FallowAbandon	Whether the site includes fallow/abandoned fields (“1”) or not (“0”) within a 75‐m radius from the centre of the site	
CultiField	Whether the site includes vegetation/grain fields (“1”) or not (“0”) within a 75‐m radius from the centre of the site	
Pasture	Whether the site includes pastures (“1”) or not (“0”) within a 75‐m radius from the centre of the site	
Lotus	Whether the site includes lotus fields (“1”) or not (“0”) within a 75‐m radius from the centre of the site	
Paddy	Whether the site includes rice paddies (“1”) or not (“0”) within a 75‐m radius from the centre of the site	
Waterbody.p1	Proportion of open waterbodies (river, stream, or lake) within a 500‐m buffer	Range: 0.0–1.0
Species columns	Individual counts of each species at each study site	*n*

As used in the previous study (Hisano et al. 2025), we further measured open waterbody areas within a 500 m buffer radius, based on previous studies (Chan et al. 2007; Amano et al. 2008), from the center of each plot by QGIS software. The buffer scale of 500 m was selected based on Hisano et al. (2025), which demonstrated that this radius effectively captures patterns of bird diversity and abundance in the study area. Similar spatial scales have also been effective in agricultural wetlands throughout East Asia (Chan et al. 2007; Amano et al. 2008; Amira et al. 2018; Lee and Goodale 2018). We then calculated the proportion of the waterbody area by dividing it by the total buffer area (78.5 ha). We obtained land‐use data from the 6th (1999–2012) and 7th (2013–) National Surveys on the Natural Environment (scale 1:25,000) available from the Ministry of the Environment, Government of Japan (https://www.biodic.go.jp/kiso/vg/vg_kiso.html).

### 2.4 Data Structure

#### 2.4.1 Data Files

We provide two types of bird species assemblage datasets. The first (“all_data.csv”) includes all observed birds, including those flying over the plot. The second (“ground_data.csv”) excludes flyovers and includes only individuals observed on the ground or perching on trees or electric wires, confirming their use of the habitat. These datasets include the information of coordinates, survey dates and time, species names, and individual counts, presence/absence of specific land‐use types (or crop types) within a 75 m radius, and the proportion of open waterbodies within a 500 m radius buffer (see Table 2).

#### 2.4.2 File Format

The data were comma‐delimited (UTF‐8).

#### 2.4.3 Variable Definitions

See the definitions provided in Table 2.

#### 2.4.4 Unit Definitions

See the definitions provided in Table 2.

### 2.5 Accessibility

#### 2.5.1 License

CC BY 4.0.

#### 2.5.2 Location of Storage

The datasets are archived in *figshare*: https://doi.org/10.6084/m9.figshare.29073710.v3.

## Author Contributions

**Masumi Hisano:** conceptualization (lead), data curation (lead), formal analysis (lead), funding acquisition (lead), investigation (lead), methodology (equal), project administration (lead), resources (lead), validation (lead), visualization (lead), writing – original draft (lead), writing – review and editing (lead). **Ken Motomura:** investigation (supporting), writing – review and editing (supporting). **Keinosuke Sannoh:** data curation (supporting), writing – review and editing (supporting). **Shota Deguchi:** investigation (supporting), methodology (equal), writing – review and editing (supporting).

## Conflicts of Interest

The authors declare no conflicts of interest.

## Supporting information


**Data S1:** ece372039‐sup‐0001‐Supinfo.docx.

## Data Availability

The datasets are archived in figshare: https://doi.org/10.6084/m9.figshare.29073710.v3.
