# Supplementary material for: Datasets of Bird Species Composition in a Land Reclamation Area of Lake Kahokugata, Central Japan, in Relation to Various Farmland Types
Source: Ecol Evol. 2025 Aug 19;15(8):e72039. doi: 10.1002/ece3.72039 (PMC12364527; doi:10.1002/ece3.72039)
Supplement: Supplementary file 1 — Data S1: ece372039‐sup‐0001‐Supinfo.docx. [file ECE3-15-e72039-s001.docx]

Supplementary Information for

**Datasets of bird species composition in a land reclamation area of Lake Kahokugata, central Japan, in relation to various farmland types**

Hisano et al.

^*^Correspondence: [hisano@hiroshima-u.ac.jp](mailto:hisano@hiroshima-u.ac.jp)

**This file includes:**

- Supplementary Text
- Supplementary Figure
- Reference for Supplementary Information

# Supplementary Text

We also obtained land use data of forested areas, grasslands, cultivated areas, urbanised areas, abandoned lands, and pastures of the study area from the 6th (1999–2012) and 7th (2013–) National Surveys on the Natural Environment (<https://www.biodic.go.jp/kiso/vg/vg_kiso.html>). Ideally, these variables could have been included in our dataset. However, the land reclamation area of Lake Kahokugata exhibits a highly heterogeneous landscape (see <http://iida.yupapa.net/sien/basic/agri/field/field.htm>), and the GIS data, which were estimated at a coarse scale, unable to accurately access this complexity. Moreover, the land use types of abandoned fields were not accurately represented in the GIS data. For example, although the landscape around site “O24” in Fig. S1 was classified as “Abandoned paddy” (see below), our field observations confirmed that it was an actively cultivated rice paddy and vice versa in some other cases. As a result, we determined that the proportion of waterbodies was the only reliable GIS-based land-use variable, and thus only this variable was included in the archived dataset ([see also Hisano et al. 2025](#_ENREF_1)). For other land use types, we supplemented the dataset with presence/absence information, which, although minimal, more accurately reflects the actual field conditions.

# Figure S1. Land use types in the study area based on the 6th (1999–2012) and 7th (2013–present) National Surveys on the Natural Environment (<https://www.biodic.go.jp/kiso/vg/vg_kiso.html>). It is important to note that this classification is not entirely accurate (see *Supplementary Text* above for details). Red circles show the locations of point-count survey sites (see *Methods*).


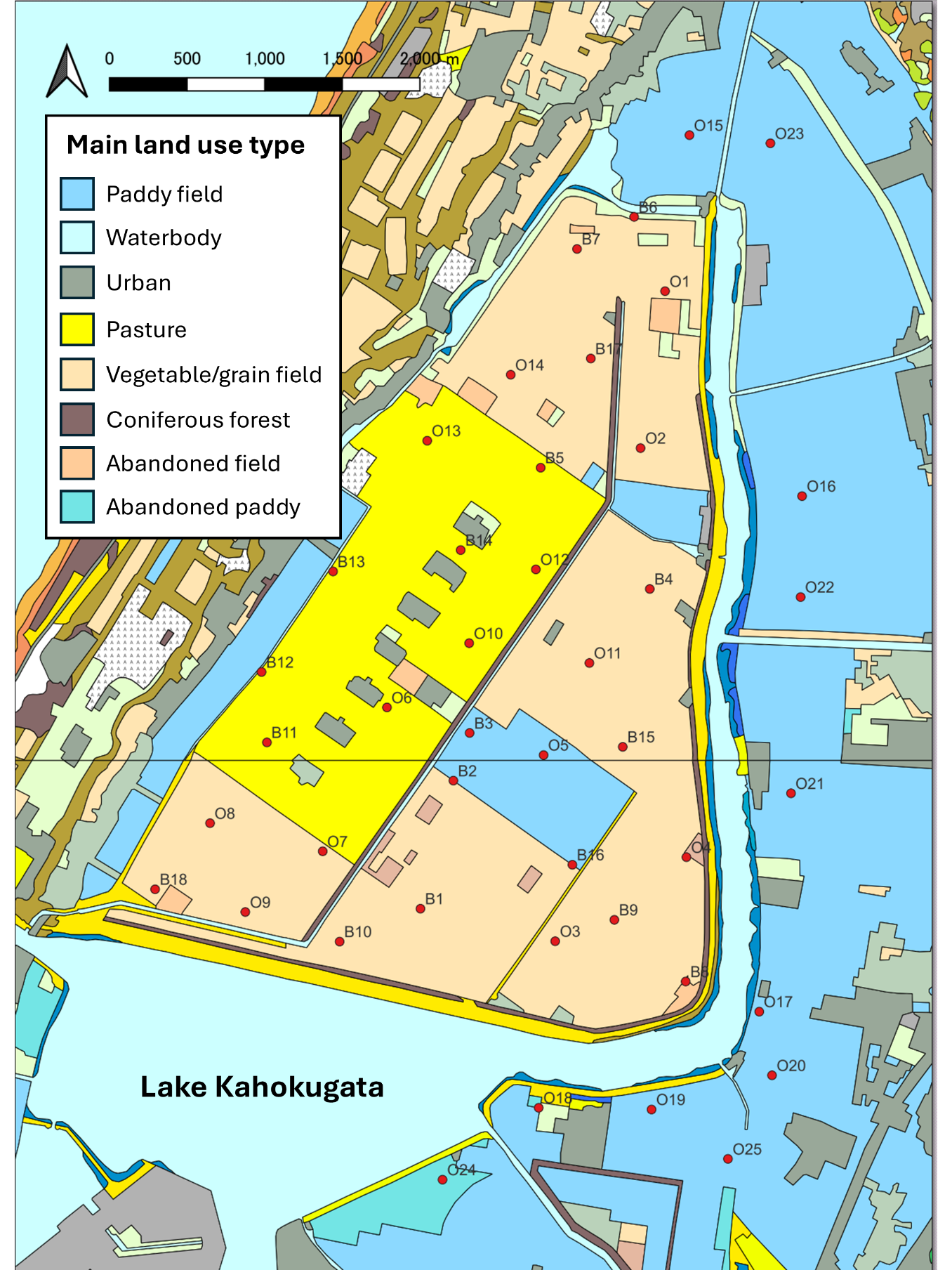


# Reference for Supplementary Information

Hisano M, Deguchi S, Sannoh K, Motomura K, Lin D-L (2025) Wetland bird utilisation of ephemerally flooded rice paddies in late winter snowmelt season in central Japan. Watershed Ecology and the Environment 7:178-186. doi: 10.1016/j.wsee.2025.04.002
